# ﻿*Podarkeopsischinensis* sp. nov. (Annelida, Hesionidae) from southeastern China

**DOI:** 10.3897/zookeys.1173.106112

**Published:** 2023-08-07

**Authors:** Shan Tong, Deyuan Yang, Jian-Wen Qiu, Caihuan Ke, Zhi Wang

**Affiliations:** 1 State Key Laboratory of Marine Environmental Science, College of Ocean and Earth Sciences, Xiamen University, Xiamen 361102, China; 2 College of Marine Science and Technology, Zhejiang Ocean University, Zhoushan 316022, China; 3 National Taiwan Ocean University, Keelung 202301, Taiwan, China; 4 College of the Environment and Ecology, Xiamen University, Xiamen 361102, China; 5 Southern Marine Science and Engineering Guangdong Laboratory (Guangzhou), Guangzhou 511458, China; 6 Department of Biology, Hong Kong Baptist University, Hong Kong, China

**Keywords:** Identification key, Indo-Pacific, molecular phylogeny, morphology, new species, polychaete, systematics

## Abstract

*Podarkeopsischinensis***sp. nov.** (Annelida, Hesionidae) is described based on specimens collected from the coast of southeast China. It is the first *Podarkeopsis* species described from the Indo-Pacific, although there are already nine valid *Podarkeopsis* species known from other parts of the world. This new species can be distinguished from the other *Podarkeopsis* species in having a palpostyle as long as the palpophore and double aciculae in both notopodia and neuropodia, and in bearing bifid furcate chaetae which have a smooth base on the shorter tine. A phylogenetic analysis based on the concatenated sequences of five gene fragments (*COI*, *16S rRNA*, *18S rRNA*, *28S rRNA*, and *histone H3*) from 18 specimens of *P.chinensis***sp. nov.** showed that they formed a monophyletic clade that is sister to *P.levifuscina*. K2P genetic distances indicated that the four gene fragments (*COI*, *16S rRNA*, *18S rRNA*, and *28S rRNA*) of *P.chinensis***sp. nov.** diverged from the corresponding sequences of the closest related species of *Podarkeopsis* in GenBank and BOLD Systems by 21.1–27.5%, 20.3–23.1%, 0.1–0.2%, and 2.1–3.2%. An identification key is provided for species in the genus *Podarkeopsis*.

## ﻿Introduction

**Figure 1.**
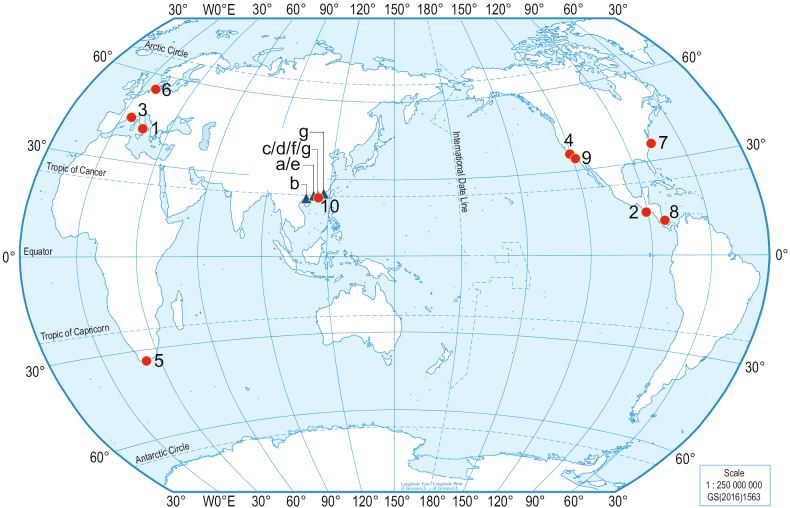
Type localities of *Podarkeopsis* species around the world (red spots and numbers, data from Read and Fauchald 2023) and records of *Podarkeopsis* from the China seas (blue triangles and letters). Numbers next to the red circles represent **1***P.arenicolus* (La Greca, 1946), Gulf of Naples **2***P.brevipalpa* (Hartmann-Schröder, 1959), El Salvador EEZ **3***P.galangaui* Laubier, 1961, Banyuls, France **4***P.glabrus* (Hartman, 1961), Southern California **5***P.capensis* (Day, 1963), South Africa **6***P.helgolandicus* (Hilbig & Dittmer, 1979), Helgoland, Germany **7***P.levifuscina* Perkins, 1984, North Carolina, USA **8***P.guadalupensis* Amoureux, 1985, Caribbean Costa Rica **9***P.perkinsi* Hilbig, 1992, California, US **10***P.chinensis* sp. nov., Daya Bay, Guangdong, China (this study). Letters corresponding to the blue triangles along southeastern China represent **a***P.* sp. A & B, Hong Kong (Shin 1998) **b***P.* sp., Beibu Gulf (Wang 2008) **c***P.galangani*, Daya Bay, Guangdong (Li 2010) **d***P.* sp., Daya Bay, Guangdong (Du et al. 2011) **e***P.* sp., Hong Kong (Wang et al. 2017) **f***P.* sp., Daya Bay, Guangdong (Zhang 2017) **g***P.chinensis* sp. nov., Daya Bay, Guangdong and ZhaoAn Bay, Fujian (this study). Source of map: No. GS(2016)1563.

*Podarkeopsis* Laubier, 1961 is a genus of small-bodied polychaetes in the family Hesionidae and currently comprises nine species (Read and Fauchald 2023), including three species from Europe, two species from the Pacific coast of the United States, one species from the Atlantic coast of the United States, one species from the Pacific coast of Central America, one species from the Atlantic coast of Central America, and one species from the Atlantic coast of South Africa (Fig. 1). Although *Podarkeopsis* species have unique morphological characteristics including having 10 triangular papillae, a pair of palps and three antennae placed towards the anterior prostomial margin, and eight pairs of tentacular cirri, its species diversity and phylogenetic relationships remain poorly understood, partially due to their minute size (a complete specimen is 5–18 mm long, 1–2 mm wide, and with 25–46 chaetigers), making them difficult to collect.

Currently, there are only a few studies with a description or specimen records of *Podarkeopsis* from Indo-Pacific. Imajima (2007) described *P.brevipalpa* (Hartmann-Schröder, 1959) and *P.glabrus* (Hartman, 1961) based on specimens collected from Japanese waters, which is the only morphological record of *Podarkeopsis* in the Indo-Pacific. However, this record is questionable, as the type locality of *P.brevipalpa* is the coast of El Salvador in Central America, and the type locality of *P.glabrus* is in southern California (Fig. 1). In addition, *Podarkeopsis*, mostly identified to the genus level, has been recorded in several ecological studies in the China seas (Shin 1998; Wang 2008; Li 2010; Du et al. 2011; Wang et al. 2017; Zhang 2017). *Podarkeopsisgalangani* (a misspelling of *P.galangaui* Laubier, 1961) has been reported from Daya Bay, Guangdong, China, but this record is questionable, given that its type locality is the Mediterranean coast of France (Li 2010).

In this study, we describe and illustrate a new species, *Podarkeopsischinensis* sp. nov., based on specimens collected from Daya Bay, Guangdong and ZhaoAn Bay, Fujian in southern China. We sequenced five gene fragments (*COI*, *16S rRNA*, *18S rRNA*, *28S rRNA*, and *histone H3*) to determine the phylogenetic position of the new species within *Podarkeopsis*.

## ﻿Material and methods

### ﻿Sample collection and preservation

Eighteen specimens (holotype: XMU-Pol-2021-105, paratype 1: XMU-Pol-2021-106, paratype 2 MBM287621, paratype 3: XMU-Pol-2021-197, paratype 4: XMU-Pol-2021-201, paratype 5: XMU-Pol-2021-203, paratype 6: XMU-Pol-2021-204, paratype 7: XMU-Pol-2021-205, paratype 8: XMU-Pol-2021-207, paratype 9: XMU-Pol-2021-208, paratype 10: XMU-Pol-2021-209, paratype 11: XMU-Pol-2021-213, paratype 12: XMU-Pol-2021-214, paratype 13: XMU-Pol-2021-215, paratype 14: MBM287622, paratype 15: XMU-Pol-2021-221, paratype 16: XMU-Pol-2021-223, paratype 17: XMU-Pol-2021-224) were collected from the coastal waters of southeast China in 2021 (see Materials examined for details). Sediments were sorted with a 0.5 mm sieve, and the retained materials including the specimens were anaesthetized with 7% MgCl_2_ solution, transferred to 50% ethanol for preliminary fixation, and then to 100% ethanol for final fixation. *Podarkeopsis* specimens were picked out under a stereomicroscope M165C in the laboratory, preliminarily identified to species, and preserved for further morphological and molecular analyses. Two specimens of *P.chinensis* sp. nov. (paratype 2, paratype 14) were deposited in the Marine Biological Museum, Chinese Academy of Sciences (MBMCAS), and the other 16 specimens of *P.chinensis* sp. nov. (holotype, paratypes 1, paratypes 3–13, and paratypes 15–17) were deposited in the specimen collections of the College of Ocean and Earth Sciences, Xiamen University (XMU).

### ﻿Morphological analysis

Selected parapodia of the holotype (XMU-Pol-2021-105), paratype 10 (XMU-Pol-2021-209) and paratype 12 (XMU-Pol-2021-214) were dissected with iridectomy scissors and permanently mounted on slides for observation of their gross morphology and chaetae, as well as for photography. To observe the minute teeth inside the pharynx, the anterior region of the paratype 6 (XMU-Pol-2021-204), paratype 11 (XMU-Pol-2021-213), and paratype 13 (XMU-Pol-2021-215) were hyalinized with graded series of glycerol (30%, 60%, 100%), mounted on slides and compressed slowly with a cover glass. Photographs of the whole specimen and parapodia (with chaetae) were taken using a camera DMC5400 mounted on a Leica M165C stereomicroscope. Photographs were taken at different focuses and stacked into fully focused images using Helicon Focus v. 7 as described by Wang et al. (2018). The anterior region of the paratype 5 (XMU-Pol-2021-203) and paratype 12 (XMU-Pol-2021-214) were treated by critical point drying and fixed on a conductive adhesive for gold plating. Photographs of the anterior end were taken using Phenom ProX scanning electron microscope (SEM). The classification of the type of parapodia following Jarvis (2011): biramous with many emergent notochaetae; sub-biramous with few emergent notochaetae; sesquiramous with acicula inside cirrophore and without emergent chaetae.

### ﻿DNA extraction, PCR amplification, and sequencing

Eighteen specimens of *Podarkeopsischinensis* sp. nov. were used for DNA extraction. For each specimen, a few segments were dissected, and genomic DNA was extracted with a DNeasy Blood & Tissue Kit (QIAGEN). Five primer pairs were used to amplify corresponding gene fragments, viz., PolyLCO and PolyHCO for the mitochondrial *COI* gene fragment (Carr et al. 2011), 16SAR-L and 16SBR-H for the mitochondrial *16S rRNA* gene fragment (Palumbi et al. 1991), 1F and 9R for the nuclear *18S rRNA* gene fragment (Glover et al. 2016); NLF184/21 and D3aR for the nuclear *28S rRNA* gene fragment (Lenaers et al. 1989; Van der Auwera et al. 1994) and H3af and H3ar for the *Histone H3* gene fragment (Colgan et al. 1998). The PCR protocol followed Zhang et al. (2018). The PCR products were checked by electrophoresis in a 2% agarose gel, and sequenced using Sanger sequencing at Xiamen Borui Biological Technology Co., Ltd.

### ﻿Phylogenetic analyses

The sequences of the five gene fragments generated in this study, together with those of all corresponding *Podarkeopsis* and two *Oxydromus* species (outgroup) available in GenBank (https://www.ncbi.nlm.nih.gov/Genbank) and BOLD (http://www.barcodinglife.org), were used for phylogenetic analyses (Table 1). The five gene sequences were aligned using the MUSCLE algorithm (Edgar 2004). The poorly aligned positions were removed with the Gblocks v. 0.91b plugged in PhyloSuite v. 1.2.3 (Zhang et al. 2020). Phylogenetic analyses were conducted using the maximum-likelihood (ML, IQ-TREE v.2.2.0 plugged in PhyloSuite) and Bayesian-inference (BI, MrBayes v.3.2.7a plugged in PhyloSuite) methods. Specifically, the ML analysis with IQ-TREE (Nguyen et al. 2015) was conducted using the “ultrafast bootstrap” option with a bootstrap number of 10,000. The best-fit evolutionary model GTR+I+G was selected for the BI analysis using ModelFinder v. 2.2.0 based on the Bayesian Information Criterion (BIC) (Kalyaanamoorthy et al. 2017). The BI analysis was conducted using MrBayes with Markov Chains run for 10,000,000 generations and topologies sampled every 1000 generations (Ronquist and Huelsenbeck 2003). The first 25% of trees were discarded as “burn-in” and the software Tracer v. 1.7.1 was used to check for the convergence of the trees (Rambaut et al. 2018). The resulting ML and BI trees were visualized using Figtree v. 1.4.4 (http://tree.bio.ed.ac.uk/software/figtree).

**Table 1.** Sequence accession numbers (GenBank and BOLD) and specimen information of *Podarkeopsis* and *Oxydromus* used in this study.

Taxon	Origin	Voucher/Sample ID	*COI*	*16S rRNA*	*18S rRNA*	*28S rRNA*	*histone H3*	Reference
*Oxydromusobscurus*	North Carolina, USA	GNM 86189	KJ855073	KJ855068	–	KJ855080	–	Martin et al. 2015
*Oxydromusmicroantennatus*	Australia	GNM 86192	KJ855072	KJ855067	–	KJ855079	–	Martin et al. 2015
*Podarkeopsisarenicolus*	France	SMNH 83509	JN571827	JN571879	JN571889	DQ442609	–	Ruta et al. 2007; Summers et al. 2015
*Podarkeopsiscapensis*	Saudi Arabia	Itsastk13-P113	KT307681	–	–	–	–	Aylagas et al. 2016
*Podarkeopsisglabrus	Washington, USA	2849_DNA	BBPS549-19	–	–	–	–	BOLD direct submission
*Podarkeopsisglabrus	Washington, USA	2852_DNA	BBPS550-19	–	–	–	–	BOLD direct submission
*Podarkeopsisglabrus	Washington, USA	2854_DNA	BBPS551-19	–	–	–	–	BOLD direct submission
*Podarkeopsisglabrus	Washington, USA	2857_DNA	BBPS552-19	–	–	–	–	BOLD direct submission
*Podarkeopsisglabrus	Washington, USA	2863_DNA	BBPS553-19	–	–	–	–	BOLD direct submission
*Podarkeopsisglabrus	California, USA	MBI-SCCWRP-00412	CMBIA476-11	–	–	–	–	BOLD direct submission
*Podarkeopsishelgolandicus*	Sweden	SE07DNA4	JN631311	–	JN631331	JN631344	–	Pleijel et al. 2012
*Podarkeopsislevifuscina*	Florida, USA	SERCINVERT2330	OQ323143	–	–	–	–	Genbank direct submission
*Podarkeopsisperkinsi*	California, USA	SIO-BIC A2339	JN571828	JN571881	JN571892	JN571901	–	Summers et al. 2015
*Podarkeopsis sp. (as *Oxydromusangustifrons*)	Laizhou Bay, Shandong, China	BIOUG03550-B01	HZPLY108-12	–	–	–	–	BOLD direct submission
*Podarkeopsis sp. (as *Oxydromusangustifrons*)	Northern Yellow Sea,China	BIOUG06836-E09	HZPLY627-13	–	–	–	–	BOLD direct submission
*Podarkeopsis sp. (as *Oxydromusangustifrons*)	Laizhou Bay, China	BIOUG06836-E11	HZPLY629-13	–	–	–	–	BOLD direct submission
*Podarkeopsischinensis* sp. nov.	Daya Bay, Guangdong, China	XMU-Pol-2021-105	MZ322693	MZ330781	OK044387	MZ344143	MZ272434	This study
*Podarkeopsischinensis* sp. nov.	Daya Bay, Guangdong, China	XMU-Pol-2021-106	MZ322694	MZ330782	OK044388	–	MZ272435	This study
*Podarkeopsischinensis* sp. nov.	Daya Bay, Guangdong, China	MBM287621	MZ873348	MZ890235	OK044406	MZ870391	MZ695068	This study
*Podarkeopsischinensis* sp. nov.	Daya Bay, Guangdong, China	XMU-Pol-2021-197	MZ873349	MZ890238	OK044409	MZ870394	MZ695069	This study
*Podarkeopsischinensis* sp. nov.	ZhaoAn Bay, Fujian, China	XMU-Pol-2021-201	MZ820673	MZ890241	OK044410	MZ820369	MZ889051	This study
*Podarkeopsischinensis* sp. nov.	ZhaoAn Bay, Fujian, China	XMU-Pol-2021-203	MZ820674	MZ890243	OK044412	MZ820371	MZ889053	This study
*Podarkeopsischinensis* sp. nov.	ZhaoAn Bay, Fujian, China	XMU-Pol-2021-204	MZ820675	MZ890244	OK044413	MZ820372	MZ889054	This study
*Podarkeopsischinensis* sp. nov.	ZhaoAn Bay, Fujian, China	XMU-Pol-2021-205	MZ820676	MZ890245	OK044414	MZ820373	MZ889055	This study
*Podarkeopsischinensis* sp. nov.	ZhaoAn Bay, Fujian, China	XMU-Pol-2021-207	MZ820677	MZ890247	OK044415	MZ820375	MZ889057	This study
*Podarkeopsischinensis* sp. nov.	ZhaoAn Bay, Fujian, China	XMU-Pol-2021-208	MZ820678	MZ890248	OK044416	MZ820376	MZ889058	This study
*Podarkeopsischinensis* sp. nov.	ZhaoAn Bay, Fujian, China	XMU-Pol-2021-209	MZ820679	MZ890249	MZ870412	MZ820377	MZ889059	This study
*Podarkeopsischinensis* sp. nov.	ZhaoAn Bay, Fujian, China	XMU-Pol-2021-213	MZ820680	MZ890253	OK044419	MZ820381	MZ889063	This study
*Podarkeopsischinensis* sp. nov.	ZhaoAn Bay, Fujian, China	XMU-Pol-2021-214	MZ820681	MZ890254	OK044420	MZ820382	MZ889064	This study
*Podarkeopsischinensis* sp. nov.	ZhaoAn Bay, Fujian, China	XMU-Pol-2021-215	MZ820682	MZ890255	OK044421	MZ870397	MZ889065	This study
*Podarkeopsischinensis* sp. nov.	ZhaoAn Bay, Fujian, China	MBM287622	MZ820683	MZ890256	OK044422	MZ820383	MZ889066	This study
*Podarkeopsischinensis* sp. nov.	ZhaoAn Bay, Fujian, China	XMU-Pol-2021-221	MZ873355	MZ890261	MZ870417	MZ870398	MZ889071	This study
*Podarkeopsischinensis* sp. nov.	ZhaoAn Bay, Fujian, China	XMU-Pol-2021-223	MZ873357	MZ890263	OK044426	MZ870400	MZ889073	This study
*Podarkeopsischinensis* sp. nov.	ZhaoAn Bay, Fujian, China	XMU-Pol-2021-224	MZ873358	MZ890264	OK044427	MZ870401	MZ889074	This study

Note: *, data from BOLD Systems; the other data were from GenBank. –, Data not available.

### ﻿K2P genetic distances

K2P genetic distances represent the standard in DNA barcoding literature and therefore facilitate comparisons (Čandek and Kuntner 2015). Intraspecific and interspecific K2P genetic distances of the five aligned gene sequences of *Podarkeopsis* species were calculated based on each gene sequence using Kimura 2-parameter (K2P) (Kimura 1980) in MEGA X. The ratio of transitions and tranversions at the first, second, and third codon positions in pairwise comparisons of aligned data set was plotted against the sequence difference values for five gene fragments (Kumar et al. 2018).

## ﻿Results

### ﻿Systematics

**Family Hesionidae Grube, 1850**

**Subfamily Ophiodrominae Pleijel, 1998**

**Tribe Ophiodromini Pleijel, 1998**

#### ﻿Genus Podarkeopsis

Taxon classificationAnimaliaPhyllodocidaHesionidae

Laubier, 1961

F23054A3-A518-515A-9194-2C3DD7E36831

##### Type species.

*Podarkeopsisgalangaui* Laubier, 1961.

##### Type locality.

Banyuls, France.

#### ﻿ Podarkeopsis chinensis sp. nov.

Taxon classificationAnimaliaPhyllodocidaHesionidae

483EEC0F-2534-56A7-B952-9D9B000A7EA3

https://zoobank.org/E34C0784-0B1E-4EE7-AF72-06F612CC5BCA

**Figure 2.**
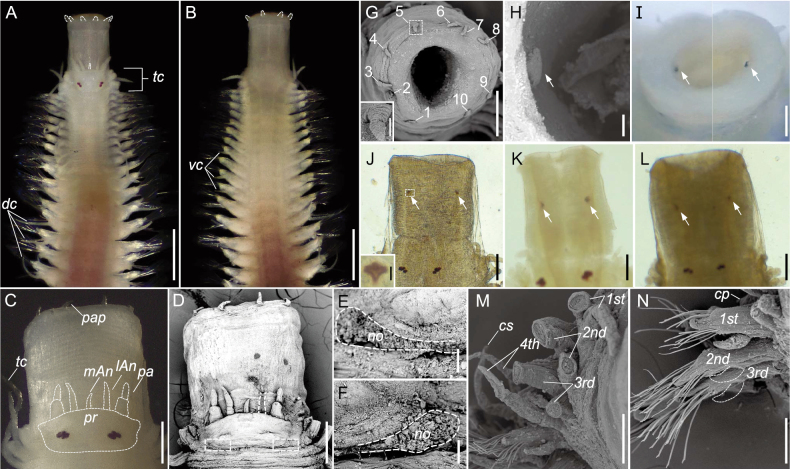
Morphology of *Podarkeopsischinensis* sp. nov. **A, B, I** holotype (XMU-Pol-2021-105) **C**–**G, M** paratype 5 (XMU-Pol-2021-203) **J** paratype 6 (XMU-Pol-2021-204) **K** paratype 11 (XMU-Pol-2021-213) **L** paratype 13 (XMU-Pol-2021-215) **H, N** paratype 12 (XMU-Pol-2021-214) **A, B** whole worm, dorsal and ventral view **C, D** anterior end, dorsal view, a dashed rectangular frame in **D** showing the position of nuchal organs **E, F** nuchal organs encircled by a dashed line **G** ring of papillae at the anterior edge of pharynx, inset: an papilla in detail **H, I** minute teeth on the inner wall of pharynx (white arrows), anterior view **J**–**L** minute teeth observed through pressed pharynx wall, dorsal view, inset in **J** showing enlarged minute tooth **M** tentacular cirri with most cirrostyles missing, right side, anterior view **N** chaetiger 1–3, right side, anterior view. Abbreviations: *pr*, prostomium; *mAn*, middle antenna; *lAn*, lateral antenna; *pa*, palp; *no*, nuchal organs; *pap*, papillae; *tc*, tentacular cirrus; *dc*, dorsal cirrus; *vc*, ventral cirrus; *cs*, cirrostyle; *cp*, cirrophore. Scale bars: 500 μm (**A, B**); 200 μm (**C, D, G, J**–**L**); 100 μm (**E, F, H**); 20 μm (**insets in G and J**).

**Figure 3.**
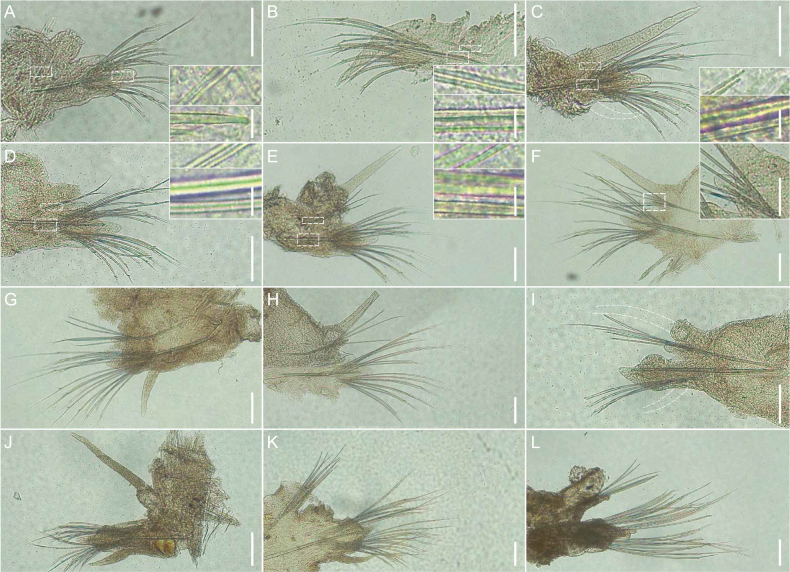
Parapodia of *Podarkeopsischinensis* sp. nov. **A–I** parapodia of holotype (XMU-Pol-2021-105) **J, K** parapodia of paratype 10 (XMU-Pol-2021-209) **L** parapodia of paratype 12 (XMU-Pol-2021-214). **A** chaetiger 1, right side, posterior view **B** chaetiger 2, right side, anterior view **C–E** chaetiger 3–5, right side, posterior view **F** chaetiger 12, right side, anterior view **G** chaetiger 13, left side, posterior view **H** chaetiger 19, left side, anterior view **I** chaetiger 20, right side, anterior view. **J** chaetiger 3, left side, posterior view **K** chaetiger 13, left side, anterior view **L** chaetiger 13, right side, posterior view. **Insets in A–E** show numbers of notopodial and neuropodial aciculae. **Insets in F** show a small notopodial lobe with five notochaetae. Scale bars: 100 μm (**A**–**L**); 10 μm (**insets in A**–**F**).

**Figure 4.**
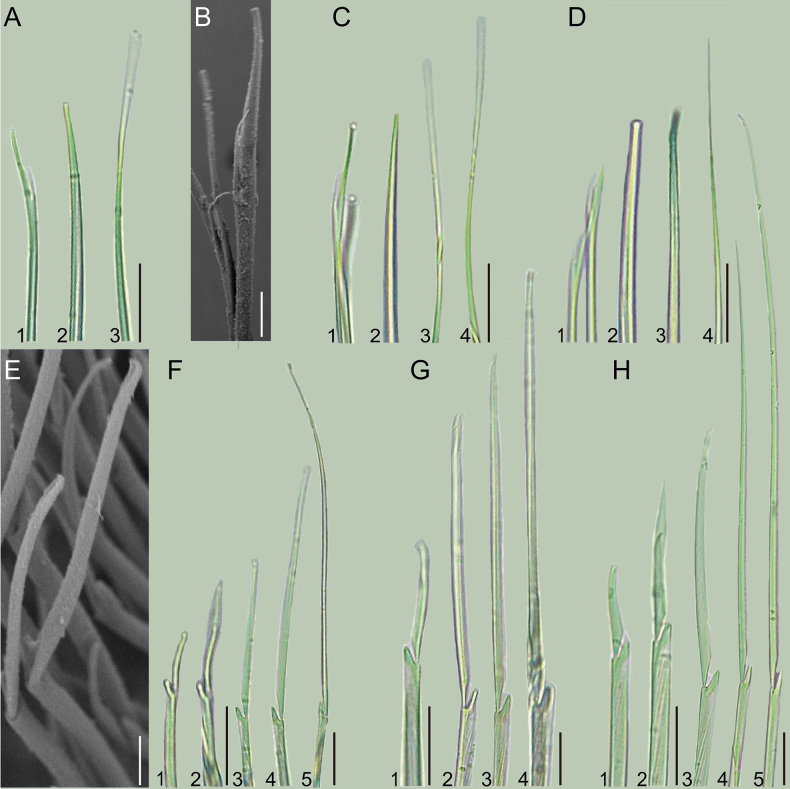
Chaetae of *Podarkeopsischinensis* sp. nov. **A, C, D, F**–**H** holotype (XMU-Pol-2021-105), light microscope; **B, E** paratype 5 (XMU-Pol-2021-203), scanning electron microscope. **A**–**D** notochaetae **A1** forked chaeta **A2** acicular chaeta **A3** capillary chaeta, chaetiger 5 **B** furcate chaetae, chaetiger 5 **C1** forked chaetae **C2** acicular chaeta **C3, C4** capillary chaetae, chaetiger 11 **D1** forked chaetae **D2, D3** acicular chaetae **D4** capillary chaeta, chaetiger 19 **E–H** neuropodial falcigers **E, F** chaetiger 1 **G** chaetiger 11 **H** chaetiger 19. Scale bars: 200 μm (**A, C, D, F3–F5, G2–G4, H3–H5**); 10 μm (**B**); 15 μm (**E**); 300 μm (**F1, F2, G1, H1, H2**).

**Figure 5.**
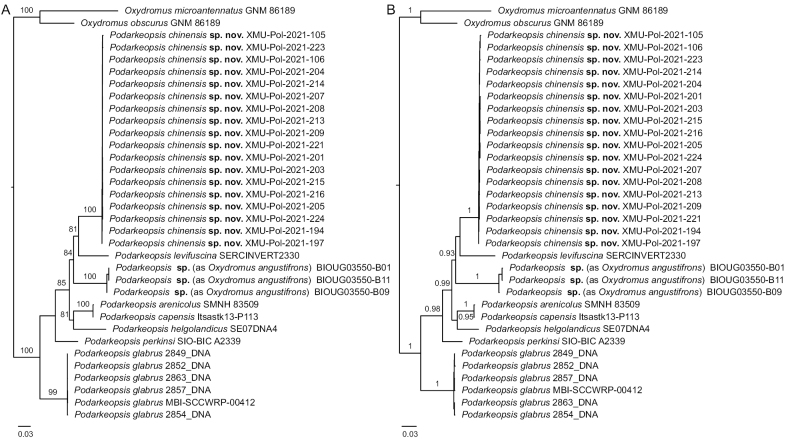
Phylogenetic trees of *Podarkeopsis* based on 3943-bp concatenated sequences of 636 bp *COI*, 496 bp *16S rRNA*, 1554 bp *18S rRNA*, 954 bp *28S rRNA* and 304 bp *histone H3* gene fragments **A** maximum-likelihood (ML) tree **B** Bayesian-inference (BI) tree. Vouchers (isolates) and accession numbers of the analyzed species are listed in Table 1. Branch support values refer to bootstrap (BS) and Bayesian posterior probabilities (BPP), and branch support values lower than 80 (BS) and 0.8 (BPP) are omitted. The scale bar indicates the number of substitutions per site.

Figs 1 , 2 , 3 , 4 , 5 , Table 1 , 2

##### Materials examined.

***Holotype***: China; Guangdong, Daya Bay, Guishan Island; 22°49'4"N, 114°47'11"E; 1 April 2021; Deyuan Yang, Zhi Wang leg.; intertidal zone; XMU-Pol-2021-105, the anterior fragment with 21 chaetigers, length: 3.4 mm, width without parapodia: 0.6 mm. ***Paratypes***: China; Guangdong, Daya Bay, Guishan Island; 22°49'4"N, 114°47'11"E; 1 April 2021; Deyuan Yang, Zhi Wang leg.; intertidal zone; paratype 1 (XMU-Pol-2021-106), the anterior fragment with 16 chaetigers, length: 3.4 mm, width without parapodia: 0.6 mm. China; Guangdong, Daya Bay; 22°41'2"N, 114°37'17"E/5.6 m; 13 March 2021; Zhi Wang, Lizhe Cai, Kang Mei, Xiaoyu Zhao leg.; shallow subtidal muddy sediment; paratype 2 (MBM287621), the anterior fragment with 15 chaetigers, length: 3.0 mm, width without parapodia: 0.6 mm; paratype 3 (XMU-Pol-2021-197), the anterior fragment with 13 chaetigers, length: 2.0 mm, width without parapodia: 0.6 mm. CHINA; Fujian, ZhaoAn Bay; 23°43'14"N, 117°17'22"E/2.6 m depth; 28 May 2021; Zhi Wang, Yuyao Li leg.; shallow subtidal hard muddy sediment; paratype 4 (XMU-Pol-2021-201), the anterior fragment with 11 chaetigers, length: 1.7 mm, width without parapodia: 0.6 mm; paratype 5 (XMU-Pol-2021-203), the anterior fragment with 10 chaetigers, length: 1.4 mm, width without parapodia: 0.6 mm; paratype 6 (XMU-Pol-2021-204), the anterior fragment with 14 chaetigers, length: 2.8 mm, width without parapodia: 0.7 mm. China; Fujian, ZhaoAn Bay; 23°42'30"N, 117°18'36"E/2.6 m depth; 28 May 2021; Zhi Wang, Yuyao Li leg.; shallow subtidal hard muddy sediment; paratype 7 (XMU-Pol-2021-205), the anterior fragment with 15 chaetigers, length: 3.2 mm, width without parapodia: 0.6 mm; paratype 8 (XMU-Pol-2021-207), the anterior fragment with 14 chaetigers, length: 2.9 mm, width without parapodia: 0.6 mm; paratype 9 (XMU-Pol-2021-208), the anterior fragment with 14 chaetigers, length: 2.1 mm, width without parapodia: 0.7 mm; paratype 10 (XMU-Pol-2021-209), the anterior fragment with 16 chaetigers, length: 3.0 mm, width without parapodia: 0.6 mm. China; Fujian, ZhaoAn Bay; 23°43'11"N, 117°18'11"E/3.5 m depth; 28 May 2021; Zhi Wang, Yuyao Li leg.; shallow subtidal hard muddy sediment; paratype 11 (XMU-Pol-2021-213), the anterior fragment with 21 chaetigers, length: 3.5 mm, width without parapodia: 0.6 mm; paratype 12 (XMU-Pol-2021-214), the anterior fragment with 21 chaetigers, length: 3.9 mm, width without parapodia: 0.6 mm; paratype 13 (XMU-Pol-2021-215), the anterior fragment with 14 chaetigers, length: 3.1 mm, width without parapodia: 0.7 mm; paratype 14 (MBM287622), the anterior fragment with 2 chaetigers, length: 0.9 mm, width without parapodia: 0.5 mm. China; Fujian, ZhaoAn Bay; 23°42'34"N, 117°20'12"E/2.4 m depth; 28 May 2021; Zhi Wang, Yuyao Li leg.; shallow subtidal hard muddy sediment; paratype 15 (XMU-Pol-2021-221), the anterior fragment with 10 chaetigers, length: 1.5 mm, width without parapodia: 0.6 mm; paratype 16 (XMU-Pol-2021-223), the anterior fragment with 9 chaetigers, length: 1.4 mm, width without parapodia: 0.6 mm; paratype 17 (XMU-Pol-2021-224), the anterior fragment with 10 chaetigers, length: 1.5 mm, width without parapodia: 0.6 mm.

##### Diagnosis.

Two pairs of eyes arranged in a trapezoid shape. Palps one pair, biarticulated, palpostyle as long as the palpophore. Double aciculae in both notopodia and neuropodia. Notopodial furcate chaetae present, base of the shorter tine smooth.

##### Description

(based on holotype, unless otherwise stated). Anterior fragment with 1–21 chaetigers. Body cylindrical. Fixed specimens uniformly pale (Fig. 2A, B).

Prostomium twice as wide as long. Eyes two pairs, placed towards the posterior prostomial margin, trapezoidally arranged, anterior pair kidney-shaped, larger than posterior pair, posterior pair oval (Fig. 2A, B). Palps one pair, biarticulated, palpostyle as long as palpophore. Three antennae, tapered. Median antenna thinner than lateral ones, lost in holotype, about half the length of lateral ones in paratype 5 (Fig. 2A, C, D). Nuchal organs of paratype 5 on lateral-posterior edge of prostomium (Fig. 2E, F). Pharynx strong, reversible, anterior edge with 10 triangular papillae (Fig. 2A, B, G). Teeth, minute, one pair, nearly triangular, symmetrically distributed on the inner wall of pharynx, visible from anterior view of pharynx in both holotype and paratypes (Fig. 2H–L). Tentacular cirri eight pairs, biarticulated, most cirrostyles missing (Fig. 2A, B).

Parapodia sesquiramous with acicula in cirrophore and without protruding notopodial chaetae in chaetigers 1–3 (Fig. 3A–C), biramous thereafter (Fig. 3D–I). Notopodial aciculae one pair, extending into cirrophores in chaetigers 1–4, extending into a small notopodial lobe in following chaetigers (Fig. 3A–I). Notopodial cirri digitate, about twice the length of neuropodial lobe, neuropodial cirri thinner, not longer than neuropodial lobe (Fig. 3C, E–G, J). Neuropodial aciculae one pair, neuropodial lobe in anterior chaetigers developed, prechaetal lobe long, digitated, postchaetal lobe rounded (Fig. 3A–F).

Notochaetae 3 types. Furcate chaetae 2 or 3, bifid, base of the shorter tine smooth, longer tine about 2.3 times as long as shorter one (Fig. 4A–D). Acicular chaetae blunt, 1 or 2 (Fig. 4A, C, D). Capillary chaetae smooth and slender, longer than furcate chaetae and acicular chaetae, 1 or 2 in number (Fig. 4A, C, D). Neurochaetae all composite falcigers (Fig. 4E–H). Blade of all falcigers unidentate, blade length/width ratios ranging from 7 to 80 (Fig. 4F–H). Hooded neurochaetae rarely present, 0 or 1 per parapodia; if present, in subacicular bundle of neurochaetae (Fig. 4F2, H2).

##### Intraspecific variation.

Examination of the holotype and several paratypes of *P.chinensis* sp. nov. revealed different numbers of the three kinds of notochaetae. In the holotype, there were 2 or 3 furcate chaetae, 1 or 2 acicular chaetae, and 1 or 2 capillary chaetae. However, several paratypes had 2–4 furcate chaetae, 1–4 acicular chaetae, and 1–3 capillary chaetae. The number of these chaetae may be related to the developmental stages or environmental conditions.

##### Remarks.

The new species can be distinguished from the other nine species of the genus in having 1) median antenna about half as long as lateral ones, while *P.arenicolus*, *P.brevipalpa*, *P.galangaui*, *P.glabrus*, and *P.helgolandicus* have median antenna shorter than half of the lateral ones (Hartmann-Schröder 1959; Hartman 1961; Laubier 1961; Hilbig and Dittmer 1979; Rizzo and Salazar-Vallejo 2014); another species, *P.perkinsi*, however, has median antenna about two-thirds as long as lateral one (Hilbig 1992); 2) ventral cirri as long as, or barely shorter than neuropodial lobe, while *P.brevipalpa*, *P.galangaui*, and *P.glabrus* have ventral cirri apparently longer than neuropodial lobe (Hartmann-Schröder 1959; Hartman 1961; Laubier 1961), *P.guadalupensis* and *P.levifuscina* have ventral cirri markedly shorter than neuropodial lobe (Amoureux 1985; Perkins 1984); 3) double aciculae in both notopodia and neuropodia, while *P.capensis*, *P.glabrus*, and *P.perkinsi* have one aciculae in both notopodia and neuropodia (Day 1963; Hartman 1961; Hilbig 1992), and *P.levifuscina* has one acicula in notopodia and double aciculae in neuropodia (Perkins 1984); and 4) notopodial furcate chaetae with both handles smooth, while *P.galangaui*, *P.glabrus*, *P.guadalupensis*, *P.helgolandicus*, and *P.perkinsi* have notopodial furcate chaetae with denticulated at the base of either the shorter handle or both handles (Hartman 1961; Laubier 1961; Hilbig and Dittmer 1979; Amoureux 1985; Hilbig 1992).

##### Etymology.

The specific name *chinensis* is an adjective in the nominative singular, derived from China, where the specimens were collected. The suggested formal Chinese name for this species is “中国健足虫”.

##### Habitat.

Intertidal, shallow subtidal muddy sediment.

##### Distribution.

*Podarkeopsischinensis* sp. nov. is currently known from Daya Bay, Guangdong and ZhaoAn Bay, Fujian, China. It is expected that this species is widely distributed along the coast of southeast China.

### ﻿Phylogenetic analysis

The ML tree and BI tree, reconstructed based on the 3943-bp concatenated sequences, showed consistent topologies clustering the eight analyzed *Podarkeopsis* species within a single clade with high support values (BS = 100; BPP = 1) (Fig. 5). All 18 specimens of *Podarkeopsischinensis* sp. nov. were clustered within a clade with high support values (BS= 100, BPP = 1), and *P.chinensis* sp. nov. was sister to *P.levifuscina* collected from Florida, USA (BS = 79; BPP = 0.84). The clade comprising the two species was sister to *Podarkeopsis* sp. (as *Oxydromusangustifrons* Grube, 1878) from Bohai Sea, China (BS = 86; BPP = 0.88). The results of phylogenetic analyses indicated *P.chinensis* sp. nov. could be distinguished as a new species by its clustering relationship with high support.

### ﻿K2P genetic distances

The average intraspecific K2P genetic distances of *Podarkeopsischinensis* sp. nov. were 0.2% for *COI* and *16S rRNA* gene fragments, 0.0% for *18S rRNA* gene fragment, 0.1% for *28S rRNA* gene fragment, and 0.5% for *histone H3* gene fragment (Table 2). Besides, the interspecific genetic distances between *P.chinensis* sp. nov. and the other *Podarkeopsis* species ranged from 21.1–27.5% for *COI* gene fragment, 20.3–23.1% for *16S rRNA* gene fragment, 0.1–0.2% for *18S rRNA* gene, and 2.1–3.2% for *28S rRNA* gene fragment.

**Table 2.** Intraspecific and interspecific K2P genetic distances of the five gene fragments of all available *Podarkeopsis* species.

Species	*N*	Species							
		1	2	3	4	5	6	7	8
***COI* (585 bp)**									
1. *Podarkeopsiarenicolus*	1	–							
2. *Podarkeopsicapensis*	1	0.005	–						
3. *Podarkeopsiglabrus*	6	0.221	0.235	0.003					
4. *Podarkeopsihelgolandicus*	1	0.227	0.224	0.265	–				
5. *Podarkeopsilevifuscina*	1	0.232	0.230	0.250	0.240	–			
6. *Podarkeopsiperkinsi*	1	0.227	0.223	0.233	0.237	0.227	–		
7. *Podarkeopsis* sp. (as *Oxydromusangustifrons*)	3	0.218	0.223	0.258	0.227	0.225	0.250	0.009	
8. *Podarkeopsichinensis* sp. nov.	18	0.271	0.264	0.275	0.271	0.211	0.246	0.252	0.002
***16S rRNA* (389 bp)**									
1. *Podarkeopsiarenicolus*	1	–							
2. *Podarkeopsiperkinsi*	1	0.192	–						
3. *Podarkeopsichinensis* sp. nov.	18	0.203	0.231	0.002					
***18S rRNA* (854 bp)**									
1. *Podarkeopsiarenicolus*	1	–							
2. *Podarkeopsihelgolandicus*	1	0.001	–						
3. *Podarkeopsiperkinsi*	1	0.002	0.001	–					
4. *Podarkeopsichinensis* sp. nov.	18	0.002	0.001	0.002	0.000				
***28S rRNA* (682 bp)**									
1. *Podarkeopsiarenicolus*	1	–							
2. *Podarkeopsihelgolandicus*	1	0.019	–						
3. *Podarkeopsiperkinsi*	1	0.032	0.028	–					
4. *Podarkeopsichinensis* sp. nov.	18	0.021	0.023	0.032	0.001				
***histone H3* (284 bp)**									
1. *Podarkeopsichinensis* sp. nov.	18	0.005							

## ﻿Discussion

Although *Podarkeopsis* is a common genus of polychaete in intertidal and subtidal sediments, it has attracted little attention, possibly due to its small size, fragilility, and usually incomplete condition of fixed specimens. This genus can be clearly distinguished from other hesionid genera by several remarkable characteristics, such as having the pharynx with 10 papillae on the anterior edge (vs no papillae as in *Oxydromus*, *Hesione*, etc.; ~20 papillae as in *Micropodarke*) and bearing four pairs of tentacular cirri on both sides of the peristomium (vs three pairs as in *Oxydromus*, *Micropodarke*, *Syllidia*, etc.) (Pleijel 1998; Rizzo and Salazar-Vallejo 2014). Therefore, it is not difficult to identify specimens to the genus level.

The genus *Podarkeopsis* currently includes only nine valid species, and in this study we describe a tenth species, *P.chinensis* sp. nov., the first *Podarkeopsis* species described from the Indo-Pacific. *Podarkeopsiscincinnata* (Verrill, 1881) collected from New England, USA, has three pairs (instead of four as in other species of *Podarkeopsis*) of slender tentacular cirri on each side and about 12 papillae according to the original description; therefore, it is considered here an invalid species of *Podarkeopsis*, and its status could not be determined without checking the type material. In addition to *P.chinensis* sp. nov., we also found some other species of *Podarkeopsis* from the China seas, which indicated an underestimated species diversity of this genus. Given that many undescribed species of *Podarkeopsis* and several species in this region are potentially misidentified, we predict that further studies may lead to the discovery of more species.

In addition to the mentioned characteristics, we also noted two, minute teeth on the inner wall of the pharynx in all specimens of *P.chinensis* sp. nov. examined (Fig. 2H–L), but these teeth have not been reported from the other species of *Podarkeopsis*. Besides, we observed a special type of hooded neurochaetae that had not been reported from other species of *Podarkeopsis*, but a similar kind of chaeta had been noted from some other hesionid species (Perkins 1984: 579; Wang et al. 2018: fig. 3O, P; this study, Fig. 4F2, H2), Thus, further studies should explore the use of these additional characters to distinguish species in the genus.

### ﻿Key to species of *Podarkeopsis* Laubier, 1961 (type locality given after species)

**Table:** 

1	Median antenna shorter than half of the lateral ones	**2**
–	Median antenna as long as, or longer than half of the lateral ones	**5**
2(1)	Lateral antennae as long as palps	***P.glabrus* (Hartman, 1961); California, USA**
–	Lateral antennae longer than palps	**3**
3(2)	Palpophore as long as palpostyle	***P.galangaui* Laubier, 1961; France**
–	Palpophore longer than palpostyle	**4**
4(3)	Ventral cirri markedly longer than neuropodial lobe; notopodial furcate chaetae with handle smooth	***P.brevipalpa* (Hartmann-Schröder, 1959); El Salvador**
–	Ventral cirri shorter than neuropodial lobe; notopodial furcate chaetae with handle denticulated	***P.helgolandicus* (Hilbig & Dittmer, 1979); Helgoland, Germany**
5(1)	Median antenna about 2/3 as long as lateral one	***P.perkinsi* Hilbig, 1992; California, USA**
–	Median antenna about half as long as lateral ones	**6**
6(5)	Lateral antennae longer than palps	***P.arenicolus* (La Greca, 1946); Gulf of Naples**
–	Lateral antennae as long as, or barely longer than palps	**7**
7(6)	Ventral cirri markedly shorter than neuropodial lobe	**8**
–	Ventral cirri as long as, or barely shorter than neuropodial lobe; notopodial furcate chaetae with handle smooth	**9**
8(7)	Median parapodia with double aciculae both in notopodia and neuropodia; notopodial furcate chaetae with handle denticulated	***P.guadalupensis* Amoureux, 1985; Caribbean Costa Rica**
–	Median parapodia with one acicula in notopodia and double aciculae in neuropodia; notopodial furcate chaetae with handle smooth	***P.levifuscina* Perkins, 1984; North Carolina, USA**
9(7)	Median parapodia with one acicula, both in notopodia and neuropodia; palpophore longer than palpostyle	***P.capensis* (Day, 1963); South Africa**
–	Median parapodia with double aciculae, both in notopodia and neuropodia; palpophore as long as palpostyle	***Podarkeopsischinensis* sp. nov.; Southeast coast of China**

## Supplementary Material

XML Treatment for Podarkeopsis

XML Treatment for Podarkeopsis chinensis

## ﻿Additional information

### Author ORCIDs

Shan Tong https://orcid.org/0009-0008-4206-974X

Deyuan Yang https://orcid.org/0000-0003-3735-9909

Jian-Wen Qiu https://orcid.org/0000-0002-1541-9627

Caihuan Ke https://orcid.org/0000-0002-7288-3565

Zhi Wang https://orcid.org/0000-0003-1044-9226

### Data availability

All of the data that support the findings of this study are available in the main text.
